# Influence of Blended Cements with Calcareous Fly Ash on Chloride Ion Migration and Carbonation Resistance of Concrete for Durable Structures

**DOI:** 10.3390/ma9010018

**Published:** 2016-01-02

**Authors:** Michał A. Glinicki, Daria Jóźwiak-Niedźwiedzka, Karolina Gibas, Mariusz Dąbrowski

**Affiliations:** Institute of Fundamental Technological Research, Polish Academy of Sciences, 5B Pawińskiego, Warsaw 02-106, Poland; djozwiak@ippt.pan.pl (D.J.-N.); kgibas@ippt.pan.pl (K.G.); mdabrow@ippt.pan.pl (M.D.)

**Keywords:** blended cements, calcareous fly ash, chloride migration, carbonation, durability, nuclear shielding concrete, thin sections

## Abstract

The objective of this paper is to examine the possible use of new blended cements containing calcareous fly ash in structural concrete, potentially adequate for structural elements of nuclear power plants. The investigation included five new cements made with different contents of non-clinker constituents: calcareous fly ash, siliceous fly ash, ground granulated blastfurnace slag, and a reference cement—ordinary Portland cement. The influence of innovative cements on the resistance of concrete to chloride and carbonation exposure was studied. Additionally, an evaluation of the microstructure was performed using optical microscopy on concrete thin sections. Test results revealed a substantial improvement of the resistance to chloride ion penetration into concrete containing blended cements. The resistance was higher for increased clinker replacement levels and increased with curing time. However, concrete made with blended cements exhibited higher depth of carbonation than the Portland cement concrete, except the Portland-fly ash cement with 14.3% of calcareous fly ash. The thin sections analysis confirmed the values of the carbonation depth obtained from the phenolphthalein test. Test results indicate the possible range of application for new cements containing calcareous fly ash.

## 1. Introduction

Concrete design for long-term performance in structures requires a more sophisticated approach than the prescriptive approach used for buildings and less important structures. Nuclear reactor containments must maintain high durability since they restrict the spread of radiation and radioactive contamination to the general public, which could have signfiicant consequences [1]. Among the various types of reactor containment buildings, the double-wall shell is considered to be functionally very efficient. The outer reinforced concrete wall is designed for protection against external impacts and environmental influences, and the inner pre-stressed concrete wall is designed for radiation shielding, tightness and preventing internal accidents [2]. Apart from typical service loads, the actions related to time due to “aging” of materials are carefully considered and modeled to develop the aging monitoring concept, [3]. For the long-term performance of concrete shielding structures, the protection of steel reinforcement against corrosion is of primary importance. The relevant long term deterioration mechanisms of concrete include chemical attack by CO_2_, aggressive sulphate or chloride ions. Since most concrete shielding structures are massive, limits are imposed on the temperature development in the hardening of concrete in order to prevent early age cracking. Both for a decreased risk of thermal cracking during concrete hardening and for an increased chemical resistance of hardened concrete, the use of supplementary cementitious materials is encouraged, [4]. The objective of this paper is to examine the possible use of new blended cements containing calcareous fly ash in structural concrete, potentially adequate for structural elements of nuclear power plants.

A large amount of calcareous fly ash is generated by the European power industry but its use in construction materials is quite limited. This type of ash is generated when burning lignite or brown coal. Because of the increased content of CaO, usually above 20%, its properties do not conform to the requirements of the PN-EN 450-1 [5] standard for fly ash for concrete. However, it exhibits certain pozzolanic and hydraulic properties. It was shown in [6,7,8] that, in the case of partial cement replacement with calcareous fly ash, several beneficial effects could be observed. The compressive strength of concrete was increased if the content of active silica in calcareous fly ash was higher than that in cement [6]. To overcome the issues of different gradation of ashes, several intermixtures were tried and synergy between the different types of fly ashes was found to be the main reason for the very good strength performance of the mixtures [7]. Ternary mixtures containing ordinary portland cement, 20% or 30% of calcareous fly ash (class C according to [8]), and [9] or 7% of silica fume were found to be quite effective in respect to concrete strength development and its durability indicators [10]. However, the increased variability of properties is known to impart the efficiency of calcareous fly ash in respect to compatibility with superplasticizers and stability of air entraining.

Possible use of calcareous fly ash as one of the major components of blended cements could result in several desired environmental and technical benefits, including a reduced carbon print and more consistent properties of binders. This is admissible following the common cements’ definition given in PN-EN 197-1 [11] but not exploited yet. Low carbon cements were tested at the industrial scale [12] reaching a clinker substitution factor of 50% through the use of ternary systems based on clinker, calcined clays and limestone, without compromising the performance. The alternatives studied to extend the current boundaries of clinker substitution for the production of blended cements without compromising early strength include ternary systems based on silica fume and slag [13], allowing for a significant increase in the hydration rate at an early age. The specific physical properties and chemical composition, like the grain size distribution and the content of minerals active in the presence of water and clinker, are the principal factors for selection of mineral additions of high efficiency [14,15].

In [16], the activity index of calcareous fly ash was evaluated using a relative compressive strength of mortar; and both mechanical and reological properties of Portland composite cements CEM II/A,B(S-W), CEM II/A,B(W-V) and CEM II/A,B-M(W-LL) were studied. Portland composite cements with calcareous fly ash were found to exhibit an increased water demand and to induce adverse effects on reological properties of mortar (increased flow limit and plastic viscosity) that were largely diminished after fly ash grinding. The durability study of cement mortars containing calcareous fly ash [17] revealed a positive effect on rapid chloride permeability, whereas carbonation resistance was found to decrease with increasing content of calcareous fly ash. Another study [18], not limited to mortars, revealed a reduction of air permeability coefficient of concrete by 53%, 77% and 23% for CEM II/A-W, CEM II/B-W and CEM II/B-M(S-W) cements, respectively, for w/c = 0.55. For w/c = 0.45, the coefficient kT (Torrent method) was within the limits 0.04–0.10 × 10^−16^ m^2^ (low air permeability) regardless of the type of cement.

The reviewed studies related to the use of calcareous fly ash in blended cements indicate certain potential for its application in structural concrete. Therefore, this investigation was undertaken to study the influence of new blended cements containing calcareus fly ash from brown coal combustion in “Bełchatów” power plant in Poland on the resistance of concrete to chloride ingress and carbonation.

## 2. Experimental Section

### 2.1. Materials

Calcareous fly ash from “Bełchatów” Power Plant in Poland was used to produce new blended cements. The composition of calcareous fly ash determined for a representative lot after monitoring during 18 months is given in Table 1. The specific gravity was 2.60 g/cm^3^, the fineness 46.3% and the surface area was 2400 cm^2^/g. The content of SiO_2_ above 40% is high in comparison to other calcareous fly ashes. Following the rules given by PN-EN 197-1 [11], since the content of CaO is larger than 15%, the strength of the fly ash binder should attain the compressive strength of 10 MPa. For various lots of calcareous fly ash, the compressive strength was much lower, *i.e.*, between 2 and 5 MPa. Considering the high content of active silica, this type of ash has both pozzolanic and self-hardening properties; the case when both reactive CaO and SiO_2_ are greater than 15% and 25%, respectively, seems to not be in line with the standard classification. Blended cements were manufactured by grinding Portland cement clinker together with gypsum, calcareous fly ash (W) and selected non-clinker major constituents (Table 2). Properties of siliceous fly ash (V) and ground granulated blast furnace slag (S) were in compliance with European standard requirements.

**Table 1:** Chemical composition of major constituents of blended cements by XRF method, (%).

Constituent	LOI	SiO_2_	Al_2_O_3_	Fe_2_O_3_	CaO	MgO	SO_3_	K_2_O	Na_2_O	CaO_free_
Calcareous fly ash (W)	2.12	40.88	19.00	4.25	25.97	1.73	3.94	0.14	0.13	1.07
Siliceous fly ash (V)	2.31	53.54	26.64	5.75	2.91	2.68	0.23	3.31	0.84	0.1
Blast furnace slag * (S)	1.06	1.30	6.79	1.21	45.79	5.16	2.09	0.39	0.53	-

* Glassy phase content 95.3%.

**Table 2:** The composition and physical properties of blended cements containing calcareous fly ash W (data provided by The Institute of Ceramics and Building Materials in Cracow, Poland) [18].

Type of Cement	Main constituents, %				Properties			
	Cement Clinker	Fly Ash		Slag S	Specific Gravity, (g/cm^3^)	Surface Area (Blaine), (cm^2^/g)	SO_3_ Content, (%)	Strength f_c28_ *) (MPa)
		Calcareous W	Siliceous V					
CEM I	94.5	-	-	-	3.10	3850	2.82	52.5
CEM II/A-W	80.9	14.3	-	-	3.05	3840	3.11	47.0
CEM II/B-W	67.4	28.9	-	-	2.98	3750	3.13	38.1
CEM II/B-M(V-W)	66.6	14.3	14.3	-	2.93	3750	3.13	30.9
CEM II/B-M(S-W)	66.6	14.3	-	14.3	3.03	3720	3.33	41.5
CEM V/A(S-W) **)	47.9	23.9	-	23.9	2.97	3800	3.33	31.8

*) the compressive strength determined on standard mortar specimens at 28 days; **) not defined in PN-EN 197-1 [11].

Concrete mixes were designed using natural sand 0–2 mm, crushed granodiorite aggregate 2–8 and 8–16 mm, crushed limestone aggregate 2–8 and 8–16 mm, with water-cement w/c ratio 0.55. The content of cement, the w/c ratio and the strength class C30/37 were selected to meet the limiting values for adequate chloride ions and carbonation exposure classes. The assumed slump was between 140 and 170 mm. The amount of superplasticizer (HRWR, based on polycarboxylate ether) was adjusted to maintain the target slump. The composition of prepared concrete mixes is shown in Table 3.

Standard 150 mm cube specimens were cast for compressive strength testing and cylinders with dimensions of 100 × 200 mm for chloride ion migration testing. Prisms of 100 × 100 × 500 mm were cast for carbonation testing. Until the age of 28 days, the specimens were subjected to standard moist curing conditions in a climatic chamber at a temperature of 20 °C and a relative humidity of 95%.

**Table 3:** Concrete mix design (kg/m^3^).

Concrete Series Designation	Cement		Sand 0–2 mm	Coarse Aggregate	Water	HRWR
1G	CEM I	320	630	1325 *)	176	-
2G	CEM II/A-W					0.36
3G	CEM II/B-W					0.36
4G	CEM II/B-M(V-W)					0.36
5G	CEM II/B-M(S-W)					0.36
6G	CEM V/A(S-W)					0.36
1D	CEM I	320	630	1325 **)	176	-
2D	CEM II/A-W					0.36
3D	CEM II/B-W					0.36
4D	CEM II/B-M(V-W)					0.36
5D	CEM II/B-M(S-W)					0.36
6D	CEM V/A(S-W)					0.36

*) granodiorite aggregate 2-8 mm and 8-16 mm; **) limestone aggregate 2–8 mm and 8–16 mm.

### 2.2. Test Methods

#### 2.2.1. Chloride Migration Coefficient

The chloride penetration was determined with the rapid chloride migration test described in Nordtest Method NT Build 492 [19]. Concrete specimens were subjected to external electrical potential (30 V) to force chloride ion migration. Three cylindrical specimens of diameter 100 mm were used each time, they were sliced into 50 mm high specimens and tested after 28 and 90 days of standard curing. Before testing, the side surfaces were protected against infiltration. Specimens saturated in Ca(OH)_2_ solution were placed between two chambers, one of which was filled with catholyte (10% NaCl solution) and the second with anolyte (0.3 molar solution of NaOH). Between the cathode and the anode, the electrical voltage was set to 30 V. After the measurement of current passing through the sample, the duration of the test was set. After finishing the ion migration process, the specimens were split into two parts in order to determine the depth of chloride ions’ penetration. For this purpose, the concrete section was sprayed with silver nitrate solution (0.1 M AgNO_3_) [20].

The conformity criteria for concretes according to Non-Steady State Migration Test are based on the voltage magnitude, temperature of anolite measured at the beginning and end of the test and the depth of chloride ions’ penetration, and are presented in Table 4. The non-steady-state migration coefficient, *D_nssm_*, is calculated from Equation (1) derived from the Fick’s second law:

(1) $$D_{nssm}=\frac{0.0239\;\left(273+T\right)L}{\left(U-2\right)t}(x-0.0238\sqrt{\frac{\left(273+T\right)Lx}{U-2})}$$

here:

*D_nssm_*—non-steady-state migration coefficient, (×10^−12^ m^2^/s);

*U*—absolute value of the applied voltage (V);

*T*—average value of the initial and final temperatures in the anolyte solution (°C);

*L*—thickness of the specimen (mm);

*x*—average value of the penetration depths (mm);

*t*—test duration (h).

The following evaluation of the concrete resistance to chloride ions’ penetration was applied [21]: from very good (*D_nssm_* < 2 × 10^−12^ m^2^/s) to good (2–8 × 10^−12^ m^2^/s), acceptable (8–16 × 10^−12^ m^2^/s) and unacceptable (*D_nssm_* > 16 × 10^−12^ m^2^/s).

#### 2.2.2. Carbonation Depth

After 28 days of curing in water, the concrete prisms were stored in laboratory conditions at a temperature of 21 °C until they reached a constant weight. The specimens were exposed to the atmosphere of increased CO_2_ concentration (1%) at a constant temperature (22 °C) and relative humidity (60%) in a computer-controlled CTS C −20/600/CO_2_ climatic chamber. After 0, 28, 56 and 90 days of exposure, the depth of carbonation was examined on the freshly broken surface with 1% of phenolphthalein in the solution of 70% ethyl alcohol according to PN-EN 13295:2005 [22]. The average depths of carbonation were determined.

#### 2.2.3. Compressive Strength

The compressive strength was determined with 150 mm cube specimens following the standard procedure PN-EN 12390-3:2009.

#### 2.2.4. Thin Section Analysis

Evaluation of the concrete microstructure was performed on thin sections made from concrete prisms both, before the placement in a carbonation chamber and after 56 days of exposure to 1% CO_2_. The fluorescent epoxy impregnated thin sections used for this study were prepared according to [23]. The concrete specimens were cut in small blocks (40 × 50 mm) in such a way that they encompassed both carbonated and non-carbonated concrete areas. The blocks were vacuum impregnated using a low viscous resin with yellow fluorescent dye. The final thin section made from the fully impregnated concrete had thickness of 20–25 μm. Thin section analysis were prepared using optical polarizing microscope Olympus BX51 (Olympus, Tokyo, Japan) connected with a digital camera. The thin sections were examined in plane polarized light (PPL), crossed polarized light (XPL), and also with lambda plate and UV light.

## 3. Test Results

### 3.1. Compressive Strength and Chloride Permeability

The compressive strength of concrete specimens ranged from 34.3 to 52.4 MPa (Table 4). The highest compressive strength values were obtained for concrete containing CEM II/A-W cement, which were about 10% higher than the strength of reference concrete containing CEM I. The lower values of the 28-days compressive strength *f_c_* < 40 MPa were attained for concretes with the highest clinker substitution rate CEM V/A(S-W) and a (V-W) combination with limestone aggregate. Concrete specimens made with blended cements, which contained both calcareous fly ash and other mineral additives, showed lower compressive strength for limestone aggregate than for granodiorite aggregate. Such differences were within the limits of 3%–17%.

**Table 4:** Concrete compressive strength after 28-days of curing (average values of three specimens).

Cement Type	Compressive Strength, *f_c28_* (MPa)	
	Granodiorite Aggregate	Limestone Aggregate
CEM I	48.5	46.8
CEM II/A-W	52.1	52.4
CEM II/B-W	47.1	48.6
CEM II/B-M(V-W)	41.5	34.3
CEM II/B-M(S-W)	50.0	43.5
CEM V/A(S-W)	39.6	36.7

**Figure 1:**
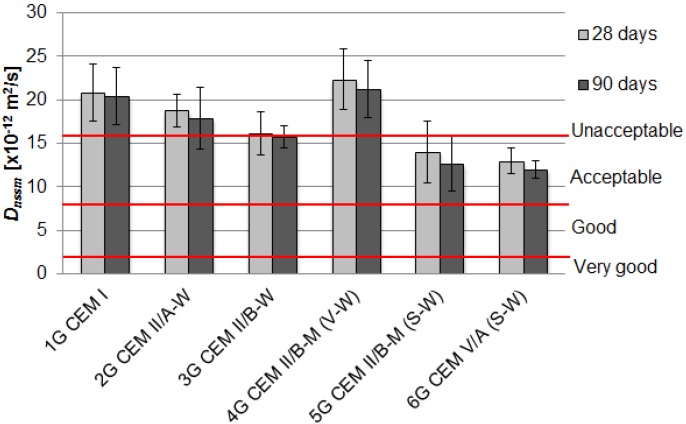
The influence of type of cement on the chloride migration coefficient *D_nssm_* for concrete with granodiorite aggregate.

**Figure 2:**
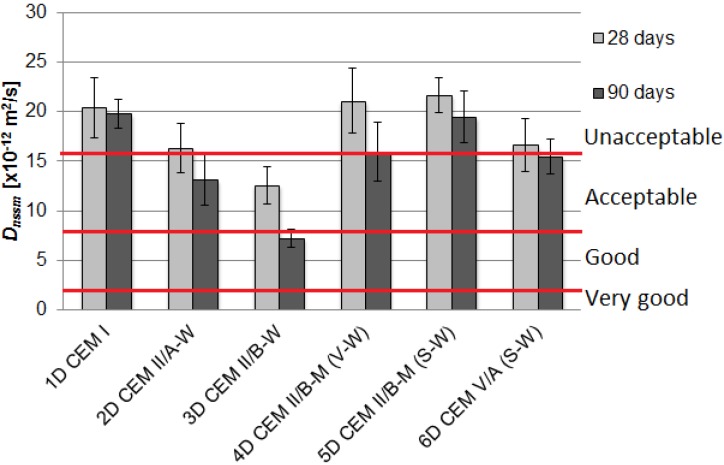
The influence of type of cement on the chloride migration coefficient *D_nssm_* for concrete with limestone aggregate.

The values of chloride migration coefficient *D_nssm_* after 28 and 90 days of curing are presented in Figure 1 and Figure 2 as the average values of results obtained with three specimens. The straight horizontal lines indicate the limits of “good”, “acceptable” and “unacceptable” resistance to chloride ions’ penetration as defined by Tang Luping [21], primarily for water and marine structures.

Beneficial effects of blended cements in concrete were observed—generally a reduction of the chloride migration coefficient was apparent. An application of cements made with calcareous fly ash and ground granulated blast furnace slag: CEM V/A(S-W) and CEM II/B-M(S-W) to concrete mixes with granodiorite aggregate was found to reduce the *D_nssm_* value by 38% and 33% in comparison with the reference concrete after 28 days of curing, and by 41% and 38% after 90 days of curing, respectively. As a result, the level of the resistance to chloride ions’ penetration increased from unacceptable to acceptable.

In the case of concrete with limestone aggregate, the best results were obtained for 2D and 3D mixes made with CEM II/A-W and CEM II/B-W containing 14.3% and 28.9% calcareous fly ash. The chloride migration coefficient after 28 days decreased by 20% and 40%, respectively. This desirable effect has yet improved over the curing time—*D_nssm_* after 90 days was reduced by 34% and 64%, respectively. The value of chloride migration coefficient concrete with CEM II/B-W was 7.21 × 10^−2^ m^2^/s and it was assigned to the concrete with good chloride ion penetration resistance. Concrete specimens containing blended cements, except for CEM II/B-M(V-W), had a higher resistance to chloride migration, as compared to the reference concrete.

Comparing the two series of concretes with different coarse aggregate, it can be concluded that the resistance to chloride migration is approximately equal for reference concrete with ordinary Portland cement and it did not improve with V-W blend in cement. The values of the *D_nssm_* were higher for the limestone aggregate than for granodiorite aggregate for ternary cements contained slag (S). The reasons could be related to differences in porosity of the interfacial transition zone. According to manufacturer’s data, the porosity of limestone aggregate was twice as much as the porosity of granodiorite aggregate, 2.1% and 1.1%, respectively. Performed mercury intrusion porosimetry tests on aggregate grains revealed further differences in pore content and pore size distribution. The relative content of large pores in the range of 100–1000 μm was 46% and 32% for limestone and granodiorite aggregate, respectively. Such grain porosity characteristics could induce noticeable differences in interfacial transition zone porosity, which resulted in greater mobility of chloride ions in concrete.

### 3.2. Rate of Carbonation

The values of carbonation coefficient *K_c_* (mm/y^1/2^) were calculated using Equation (2), based on the measurements of *x_c_* and the age of the tested specimens.

(2) $$x_{c}=K_{c}\cdot \sqrt{t}$$

where: *x_c_*—the depth of concrete penetrated by CO_2_ in mm; *t*—time of exposure in years.

**Figure 3:**
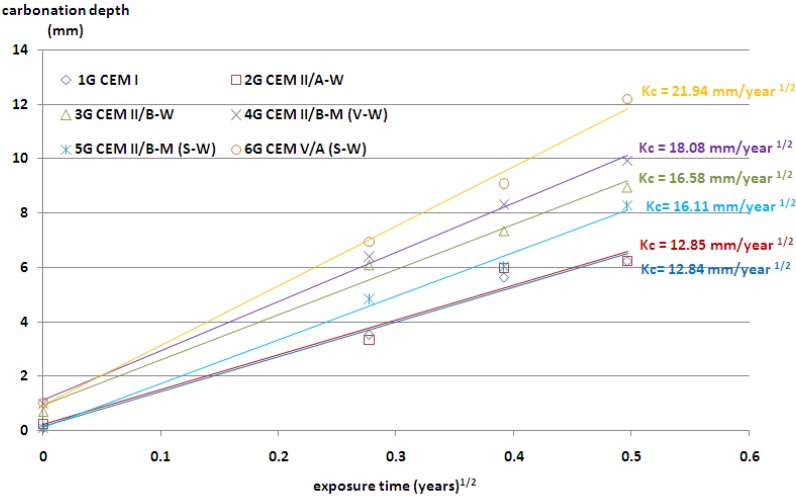
Depth of carbonation as a function of the square root of time (the values of *K_c_* coefficient); granodiorite aggregate.

**Figure 4:**
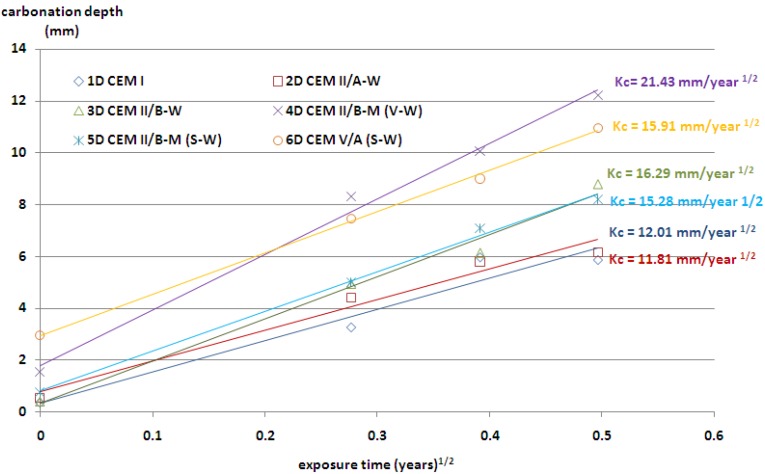
Depth of carbonation as a function of the square root of time (the values of *K_c_* coefficient); limestone aggregate.

The plot *x_c_ vs.* *t* is approximately linear and the slope is related to the *K_c_* value. The results of the rate of carbonation are presented in Figure 3 and Figure 4. As it was expected, the depth of carbonation increased, along with the age of exposure. The highest resistance to carbonation showed concretes with ordinary Portland cement, but a very similar result was obtained also for concrete with CEM II/A-W cement. The carbonation depth of about 8.5 mm was observed in concrete containing cement CEM II/B-M(S-W) and CEM II/B-W. The above results were achieved regardless of the origin of the coarse aggregate.

The lowest resistance to carbonation in concrete with granodiorite aggregate was achieved for cement containing the highest contents of non-clinker constituents—CEM V/A(S-W). The highest depth of carbonation in concretes with limestone aggregate was achieved by application of cement CEM II/B-M(V-W), with siliceous and calcareous fly ash.

The use of ternary V-W cements in concrete resulted in a slight increase in carbonation depth for medium content of non-clinker constituents. However, when the sum of V and W constituents was high, almost 50%, a major increase in carbonation depth was recorded. The same increase of carbonation depth was observed for concrete containing binary cements CEM II/A-W and CEM II/B-W. A combination of siliceous and calcareous fly ash in cement resulted in a faster carbonation and a higher carbonation depth in concrete. The frequently cited statement—that fly ash reduces the amount of calcium hydroxide which may cause an increase in the carbonation rate Papadakis [24]—is valid only for low calcium fly ash and for clinker replacement by high calcium fly ash higher than 14.3%. It was also observed that with a similar clinker factor for Portland-fly ash cement—CEM II/B-W and Portland composite cement—CEM II/B-M(V-W), the depth of carbonation was different. Probably, it was due to the different content of CaO in cement, 52.5% and 49%, respectively. The contents of portlandite or C-S-H in the relevant cement pastes are not available in order to make a proper comparison. However, the trend observed for the carbonation depth follows the differences in the compressive strength of CEM II/B-W and CEM II/B-M (V-W) cements shown in Table 2. A lower degree of reaction and related higher porosity of cement paste could be significant factors influencing the differences in carbonation rate of concrete containing these two types of cements with an equal clinker factor.

The carbonation depth in concrete progresses with exposure duration. However, the rate of carbonation reduces with respect to time period, and is usually considered as proportional to the square root of exposure period. Even in the case of accelerated carbonation conditions, the depths of carbonation are in proportion to the square root of exposure time, as in the case of natural indoor exposure conditions and natural outdoor exposure conditions under a shelter [25]. There are strong relationships between the carbonation depth of concrete tested in a natural environment and that tested in the accelerated carbonation chamber. A linear equation was proposed by Khunthongkeaw *et al*. [26] to predict the carbonation depth in natural environments based on the results obtained from the accelerated carbonation. It was assumed that the carbonation rate was approximately related to the square root of the CO_2_ concentration. According to this assumption, the predicted *K_c_* values for natural environmental conditions (CO_2_ concentration = 0.03%) are shown in Table 5.

**Table 5:** Predicted *K_c_* values for natural environmental conditions (CO_2_ concentration = 0.03%).

Cement Type	Carbonation Coefficient *K_c_* (mm/y^1/2^), Natural Conditions, CO_2_ = 0.03%	
	Granodiorite Aggregate	Limestone Aggregate
CEM I	2.24	2.09
CEM II/A-W	2.24	2.06
CEM II/B-W	2.89	2.84
CEM II/B-M(V-W)	3.15	3.73
CEM II/B-M(S-W)	2.81	2.66
CEM V/A(S-W)	3.92	2.77

The obtained results of the predicted *K_c_* values for natural environmental conditions are in good agreement with other literature data. It is know that values *K_c_* found for real structures exposed to the atmosphere but protected from rain, vary from 2 to 15 mm/y^1/2^. Indicatively, 2 < *K_c_* < 6 for concrete of low porosity (well compacted and cured, with low w/c) whose cement content is above 350 kg/m^3^; 6 < *K_c_* < 9 for concrete of medium porosity; *K_c_* > 9 for highly porous concrete with cement content below 250 kg/m^3^, [27].

### 3.3. Microscopic Evaluation of the Carbonation Depth

**Figure 5:**
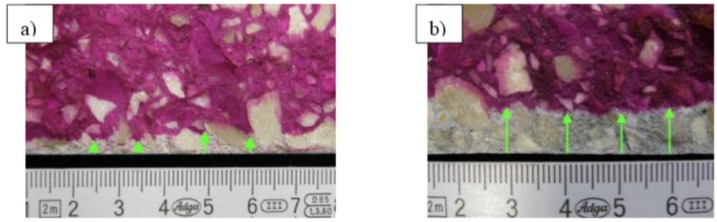
Freshly fractured concrete sprayed with phenolphthalein indicator solution after 56 days of exposition in 1% CO_2_, concrete with limestone aggregate: (**a**) CEM I; (**b**) CEM II/B-M(V-W).

**Figure 6:**
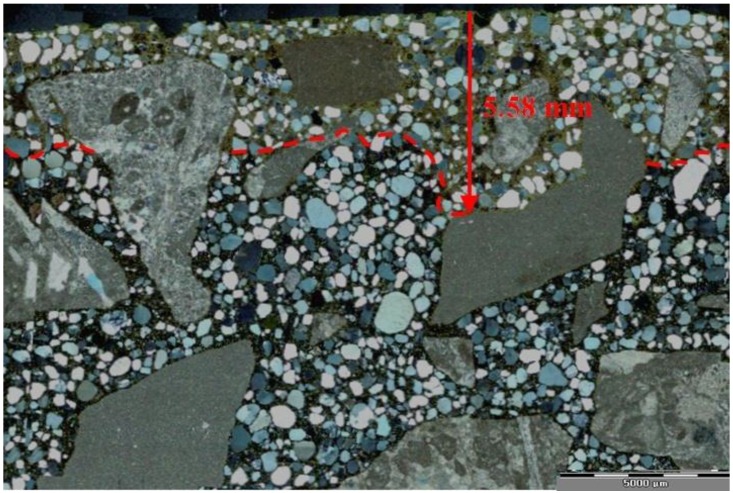
Thin-section view of concrete with limestone aggregate and CEM I with visible carbonation front, after 56 days of exposition in 1% CO_2_, XPL, scale bar = 5000 µm.

**Figure 7:**
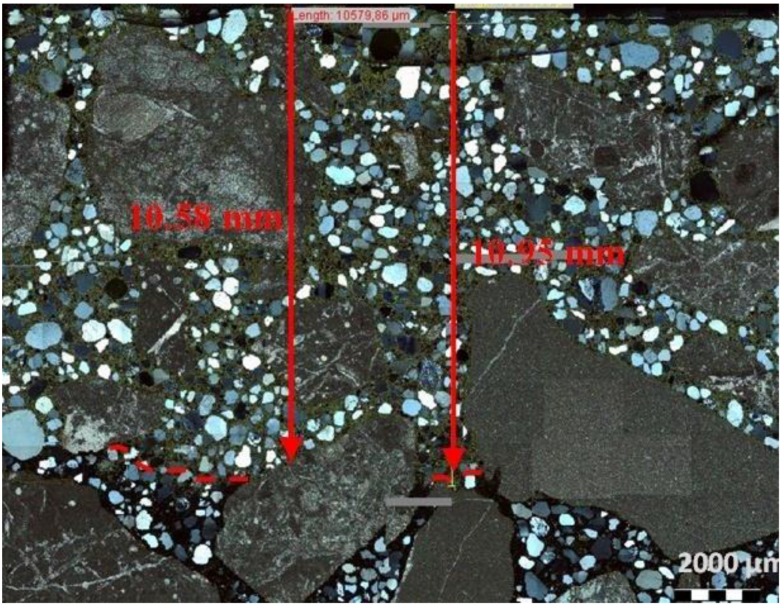
Thin-section view of concrete with limestone aggregate and CEM II/B-M (V–W) with visible carbonation front, after 56 days of exposition in 1% CO_2_, XPL, scale bar = 2000 µm.

**Figure 8:**
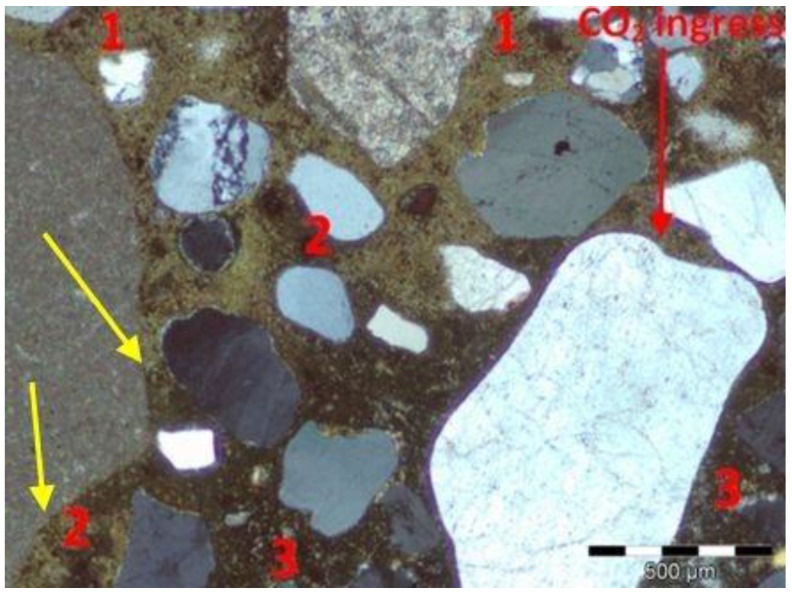
Microstructure of the partially carbonated zone in concrete with limestone aggregate and CEM I: 1—totally carbonated matrix; 2—partially carbonated matrix, 3—non-carbonated matrix; XPL, scale bar = 500 µm.

**Figure 9:**
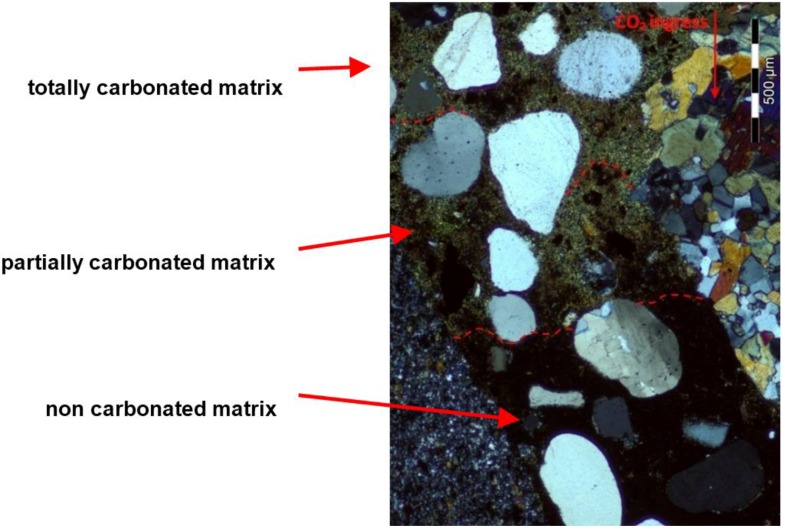
Microstructure of the three zones in concrete with granodiorite and CEM V/A (S–W); XPL, scale bar = 500 µm.

The analysis of thin sections of tested concretes confirmed the values of the depth of the carbonation obtained from the phenolphthalein test. In the uncarbonated part of the specimen where the concrete was still highly alkaline, a purple colour was obtained. In the carbonated part where the alkalinity of the concrete was reduced, no coloration occurred. Figure 5 shows examples of a cross section view of concrete with limestone aggregate with the lowest and the highest CO_2_ ingress in the cement matrix after 56 days of testing. Figure 6 and Figure 7 show, apart from coarse and fine aggregate, the cement matrix. From the top of the thin section (view in XPL), the cement matrix is much more orange-brown than in the deeper situated regions, which correspond to the carbonated matrix, and Ca(OH)_2_ has been changed into CaCO_3_. The carbonation front was evenly distributed in all tested specimens. However, a deeper carbonation front close to the limestone grain can be seen in Figure 8 as indicated by yellow arrows. This could be an indication of increased porosity in the vicinity of limestone grain. The thin section analysis showed that the depth of the partially carbonated zone, which is situated between the totally carbonated and non-carbonated zones, was similar at about 500–600 µm for all concrete series, as shown in Figure 8 and Figure 9. The differences in the depth of the partially carbonated zone due to different cement types were small. The largest depth was associated with the highest carbonation depth of 800 µm in concrete containing CEM V/A(S-W) and granodiorite aggregate.

Figure 9 shows the differences in the colour matrix analyzed on thin sections in cross-polarized light XPT in three clearly different zones; from the top: totally carbonated, partially and non-carbonated concrete matrix. It should be noted that the carbonation front is not sharp but gradual.

## 4. Discussion

The analysis of test results and a comparison with published data reveal interesting insights. A beneficial effect of calcareous fly ash in blended cements on chloride permeability is observed, whereas the carbonation resistance was found to decrease with increasing content of calcareous fly ash. A presence of active mineral admixture used as a main constituent in blended cements may inhibit permeation of aggressive media [24], and that was confirmed by this investigation in the case of calcareous fly ash. Test results revealed a substantial improvement of the resistance to chloride penetration into concrete containing blended cements with calcareous fly ash, except for the combination with siliceous fly ash CEM II/B-M(V-W). Similar results were obtained earlier for the use of calcareous fly ash as an addition to concrete mix [28,29]: the resistance was higher for increased replacement level and decreased water-to-cement ratio. Increasing resistance of concrete to the penetration of chloride ions with increasing calcareous ash content can be attributed to changes in the concrete microstructure. As it was shown in [30], the addition of calcareous fly ash reduced the content of portlandite in the matrix by 45%–74% and increased the ratio of non-evaporable water contained in cement hydration products to non-evaporable water contained in calcium hydroxide by 1.6–3.2 times. However, the effectiveness of concrete modification induced by calcareous fly ash addition is expected to be significantly influenced by the cement composition, namely the content of alite, tricalcium silicate and sulphate phases.

The use of blended cements containing calcareous fly ash decreased the resistance of concrete to carbonation at all test ages. However, when the concrete contained only 14.3% of HCFA in the cement, the carbonation depth reached the same value as for the reference concrete with Portland cement. A decreased carbonation resistance can be largely attributed to the lower calcium hydroxide content in the blended cements as suggested in [4,31].

**Figure 10:**
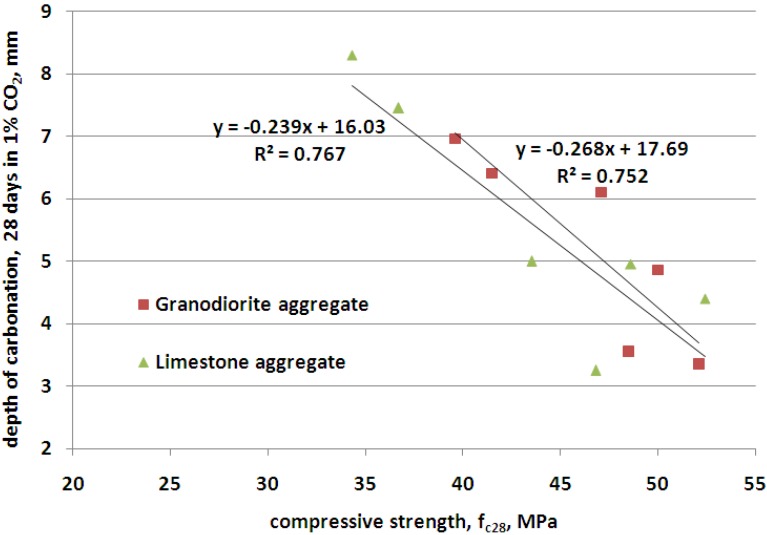
Relationship between carbonation depth of concrete and compressive strength in concretes with granodiorite and limestone aggregate.

In Figure 10, the relationship between the depth of carbonation and the compressive strength is presented. The relationship is close to linear addition, and is not much different for the two tested types of aggregates. However, such differences in the depth of carbonation are small. As suggested in [4], carbonation induced corrosion is unlikely to be a problem in properly proportioned and cured concrete, but at a relatively high clinker replacement level, it is prudent to provide additional cover, extend the moist curing period or increase the concrete strength.

The analysis of microstructure on thin sections revealed that the carbonation front is not sharp but gradual, opposite to the earlier studies and models which assume a steep carbonation front, [32]. It was confirmed by Thiéry *et al.* [33], who showed that the kinetics of the carbonation process were rate-controlling, rather than CO_2_ diffusion, which explained the gradual carbonation front.

The significance of these findings in the context of nuclear power structures is related to the high importance of these structures, their failure having large social and environmental consequences. Published data on the long term performance of containment buildings revealed many cases that required repair [34]. Overall, approximately 60% of the locations examined had deteriorated, or were repaired, as a result of chloride induced corrosion. Approximately 25% had deteriorated as a result of carbonation induced corrosion, and the remainder by drying shrinkage or other mechanisms. These were almost exclusively in locations devoid of an external source of chlorides. The carbonation was one of the main mechanisms of concrete degradation, identified on concrete cores taken from the external surface of the secondary containment building of the Tihange 2 reactor in Belgium [35]. A deeper than expected and extended carbonation front was developing on the whole structure of the containment. In general, among the major aging issues are increased permeability due to cracking or creep [2,3]. Particularly, the early age cracking can be detrimental. Therefore, a study of temperature distribution due to hydration heat release in hardening concrete containing W blended cements has also been undertaken by the authors [36]. Such a comprehensive approach is needed for a proper determination of the application range of new cements containing calcareous fly ash.

## 5. Conclusions

Concrete carbonation and chloride ingress into the cover layer are considered as the two principal factors responsible for the majority of durability issues of reinforced concrete structures designed for long-term performance. The transport of both CO_2_ and Cl^−^ occurs mostly in the pores of concrete, highlighting the significance of the pore structure which could be engineered using active mineral additions in blended cement to enhance concrete durability. An investigation into the effects of blended cements with calcareous fly ash and two types of coarse aggregates on strength, chloride ion penetration and carbonation resistance of concrete resulted in the following conclusions.

The use of new cements containing calcareous fly ash as one of the major components in concrete did not lead to any technological issues in mixing or compaction of concrete.

1 Due to pozzolanic and hydraulic activity of calcareous fly ash, the compressive strength of concrete containing blended binary cements was improved by 7% or 12% at the age of 28 days for granodiorite or limestone aggregate concrete, respectively. Increased clinker replacement levels resulted in a decrease in strength of 3%–19% or 25% for granodiorite or limestone aggregate concrete, respectively.
2 The carbonation resistance of concrete with blended cements containing calcareous fly ash increased linearly with an increase in the compressive strength of the concrete.
3 The carbonation resistance of concrete decreased with clinker replacement level, however, the depth of carbonation was low. Concrete containing Portland-calcareous fly ash cement (14.3% of calcareous fly ash) exhibited an equal carbonation resistance to a reference concrete containing CEM I.
4 The type of coarse aggregate did not affect the concrete resistance to carbonation except in the case of cement CEM II/B-M(V-W). Here, the carbonation depth of concrete containing limestone aggregate was higher by 17%–33% than for concrete containing granodiorite aggregate.
5 The concrete microstructure analysis performed on thin section confirmed the results obtained from phenoloftalein test and also revealed that the carbonation front was gradual, not sharp as previously assumed.
6 The use of cements CEM V/A(S-W) and CEM II/B-M(S-W) in concrete mixes with granodiorite aggregate resulted in the reduction of the chloride migration coefficient *D_nssm_* by 38%–41% after 90 days of curing, corresponding to an increased penetration resistance level from “unacceptable” to “acceptable”.
7 Chloride migration coefficient of concrete with limestone aggregate made with CEM II/A-W and CEM II/B-W cements decreased by 34%–64% in relation to CEM I reference concrete. The highest chloride penetration resistance, *D_nssm_* = 7.2 × 10^−12^ m^2^/s, corresponding to “good” penetration resistance, was found in the case of concrete with CEM II/B-W.
